# Progress in Medicine

**Published:** 1900-05-26

**Authors:** 


### Progress in Medicine.

### DISEASES OF THE BLOOD.

The diagnostic value of an examination of the blood is shown by the fact that it is the only tissue which can be examined chemically and microscopically during life. L. Gulland[1] remarks on the leucocytosis which occurs even from the beginning in pneumonia, and when a malignant growth recurs after removal. If leucocytosis is absent in severe cases of diseases where it is usually present a fatal termination can be foretold. It is absent normally in influenza, measles, typhoid, simple tuberculosis, and malaria. True asthma can be distinguished from renal and cardiac trouble by the increase of eosinophiles, which also marks pemphigus, and especially trichinosis. F. G. Burrows showed last year that among other states causing leucocytosis we should include ordinary convulsions. This leucocytosis is of the inflammatory type, and varies with the severity of the attacks of convulsions. A. Wright[2] mentions the poor coagulability of the blood from deficient lime salts seen in many cases of urticaria, phthisis, ununited fracture, and possibly Bright's disease. In a few cases the condition is due to deficiency of the multinuclear cells, and not to a want of lime. Here the administration of thymus tabloids may be useful; whereas, in ordinary instances, calcium chloride is the best remedy for slow coagulation. W. J. Berkeley[3] gives useful directions for staining and fixing films of blood. Thus, for detecting malarial organisms, he points out, it is much easier to use fresh than stained specimens. In staining with eosin and methylene blue trials must be made to discover the time required for the blue, which cannot be told beforehand. As short a time as possible, provided the nuclei are well stained, gives the best results as to the malarial plasmodes. In all cases the specimen should be taken as close to the time of the ague fit as possible, since examinations made on the off day give very unreliable results. Similarly, full doses of quinine may clear the organisms from the peripheral circulation in 24 hours. Hence it is not desirable to make one's diagnosis uncertain by allowing quinine to be taken before the blood has been tested.

**Pernicious Anæmia.**—W. Hunter[4] refers to his previously-expressed opinion that this disease is marked by excessive destruction of blood. This is due to a poison elaborated by some organism in the digestive tract. He now points out that stomatitis is an early symptom and is generally preceded by decay of the teeth. This suppurating matter leads to gastric catarrh and finally to a toxæmia of streptococcal type. Two groups of symptoms result—blood destruction from the poisons elaborated, and gastro-intestinal irritation from the infective catarrh which has been set up. As to treatment, he suggests the use of streptococcal serum, together with the removal of all decayed teeth, and the most perfect antisepsis possible for the stomach and bowels.

A careful record of the blood state in two cases of this disease extending over several months is given by A. C. Coles.[5] It shows the enormous reduction of the red cells, and a diminution of the hæmoglobin nearly as great, the poikilocytosis, and the presence of nucleated red cells, while the lymphocytes and eosinophiles were relatively more numerous than in normal blood. The total number of leucocytes was actually diminished in both patients, which would serve as a means of diagnosis from cases of anæmia due to malignant disease or severe hæmorrhage where the numbers would be normal or probably increased. It is interesting to note the remarkable variations in the course of this disease. At one time these patients would seem practically cured, and at another their condition would sink as rapidly. The subjective symptoms varied with the blood count, and in spite of the trial of various remedies both patients died. In view of Hunter's theory it should be added that Dr. Coles made cultures from the blood of each, but without any definite results.

In contrast with this disease, we find an account of splenic anæmia written by B. W. Sippey.[6] The enlargement of the spleen seems to be the primary change, which is followed later on by anæmia. In five cases recovery has taken place after splenectomy, while medical treatment seems quite hopeless. This patient, a man of 45, noticed the tumour three and a-quarter years before. It was followed by recurrent epistaxis and gastro-intestinal troubles, with weakness, fever, and dyspnœa. The blood count showed that the case was one of splenic anæmia, and not, as had been thought, leukæmia. At death the spleen weighed 5·2 lb.

**Leukæmia.**—Acute aural vertigo has been noticed in a few cases, as well as simple deafness, which sometimes appears in early stages of this disease. Weber, at the Royal Medico-Chirurgical Society,[7] described an instance in a man aged 31. Six months before death headache, vertigo, and vomiting came on with marked deafness. The post-mortem showed that part of the scala tympani and the peri-lymphatics of the semi-circular-canals were blocked up with fresh bony and fibroid tissue. Other cases confirm the view that hæmorrhage takes place on such occasions into the canals of both ears simultaneously. The clot is subsequently organised, and if the patient lives long enough it actually becomes ossified. At the same meeting Mott gave the history of a patient who, besides similar ear lesions and symptoms, had hæmorrhages into the posterior spinal roots, and showed inability to balance himself when walking, together with Romberg's symptom and the absence of knee-jerks. Eichhorst[8] mentions a case where paraplegic symptoms occurred from the pressure on the cord of a mass of lymphoid tissue between the fifth and seventh dorsal vertebræ. The nervous system has also been affected in leukæmia in other ways, such as by the swelling or atrophy of nerve fibres, though the instances recorded are not numerous. The effect of an infectious disease upon leukæmic patients is curious. Kormoezi[9] notes that splenic tumours and the number of the white cells are by it often diminished. The effect on the red cells is unimportant; but of the white the loss of granular cells may be made up more or less by an increase in the polynuclear ones. Th. McCrae[10] remarks on the disappearance of abnormal blood symptoms under the influence of fevers and septic infections. In other cases without such influences improvement may take place sometimes as the result of treatment, but rarely to a complete disappearance of all the symptoms. Thus the leucocytes may become normal in number, though some myelocytes or an enlargement of the spleen may remain. McCrae's own patient, however, showed a complete disappearance of all symptoms upon two occasions when under treatment by arsenic, the blood and spleen becoming normal. The leucocytes at one time had actually reached 584,000. In spite of this the fatal character of the disease was unchanged, and the patient died some months after this second apparent recovery.

[1] Brit. Med. Jour., Nov. 11. [2] Brit. Med. Jour., Jan. 5. [3] New York Med. Jour., April 21. [4] Lancet, Jan. 27. [5] Brit. Med. Jour., Mar. 31. [6] Lancet, Jan. 13. [7] Med. Rec., April 21. [8] Amer. Jour. Med. Sci. [9] Brit. Med. Jour., Jan. 13. [10] Brit. Med. Jour., Mar. 31.

